# Conjugated Oligoelectrolyte with DNA Affinity for Enhanced Nuclear Imaging and Precise DNA Quantification

**DOI:** 10.3390/bios14020105

**Published:** 2024-02-18

**Authors:** Xinmeng Zhang, Cheng Zhou, Jianxun Hou, Gang Feng, Zhourui Xu, Yonghong Shao, Chengbin Yang, Gaixia Xu

**Affiliations:** 1 Shenzhen Testing Center of Medical Devices, Shenzhen Institute for Drug Control, Shenzhen 518057, China; 2 College of Physics and Optoelectronic Engineering, Shenzhen University, Shenzhen 518060, China; 3 Institute of Polymer Optoelectronic Materials and Devices, State Key Laboratory of Luminescent Materials and Devices, South China University of Technology, Guangzhou 510640, China; 4 Guangdong Key Laboratory for Biomedical Measurements and Ultrasound Imaging, School of Biomedical Engineering, Health Science Center, Shenzhen University, Shenzhen 518060, China

**Keywords:** conjugated oligoelectrolytes, intercalative probe, nucleus imaging, DNA detection

## Abstract

Precise DNA quantification and nuclear imaging are pivotal for clinical testing, pathological diagnosis, and drug development. The detection and localization of mitochondrial DNA serve as crucial indicators of cellular health. We introduce a novel conjugated oligoelectrolyte (COE) molecule, COE-S3, featuring a planar backbone composed of three benzene rings and terminal side chains. This unique amphiphilic structure endows COE-S3 with exceptional water solubility, a high quantum yield of 0.79, and a significant fluorescence Stokes shift (λ_ex_ = 366 nm, λ_em_ = 476 nm), alongside a specific fluorescence response to DNA. The fluorescence intensity correlates proportionally with DNA concentration. COE-S3 interacts with double-stranded DNA (dsDNA) through an intercalation binding mode, exhibiting a binding constant (K) of 1.32 × 10^6^ M^−1^. Its amphiphilic nature and strong DNA affinity facilitate its localization within mitochondria in living cells and nuclei in apoptotic cells. Remarkably, within 30 min of COE-S3 staining, cell vitality can be discerned through real-time nuclear fluorescence imaging of apoptotic cells. COE-S3’s high DNA selectivity enables quantitative intracellular DNA analysis, providing insights into cell proliferation, differentiation, and growth. Our findings underscore COE-S3, with its strategically designed, shortened planar backbone, as a promising intercalative probe for DNA quantification and nuclear imaging.

## 1. Introduction

The nucleus, a critical organelle in eukaryotic cells, contains deoxyribonucleic acid (DNA), the blueprint for organismal development, growth, and reproduction [1]. It plays a regulatory role in cellular metabolism by modulating gene expression. Research in nuclear imaging and DNA quantification is vital for disease diagnosis and healthcare advancements [2]. Heckenbach et al. combined nuclear morphological studies with deep learning to accurately predict cellular senescence [3]. Clinically, quantitative DNA detection aids in the early identification of precancerous lesions [4]. Intercalative DNA probes, known for their high selectivity, sensitivity, and capability for in situ and real-time observation, have been extensively studied in nuclear imaging and DNA quantification [5]. These probes intercalate between nucleobases, causing DNA unwinding and extension of the deoxyribose-phosphate backbone, leading to changes in spectral properties such as absorbance and emission [6,7]. However, commercial probes still have limitations in use, and new intercalating probes are continuously being developed. Examples include the carcinogenic toxicity of ethidium bromide and thiazole orange [8,9,10], as well as the potential side effect of cyanine dyes causing photocleavage of DNA, especially under the intense illumination used for imaging individual molecules by fluorescence microscopy [11].

Conjugated materials have emerged as promising candidates in the realms of semi-conductive devices and biosensing, attributed to their distinctive electrical and optical properties [12,13]. Among these, conjugated oligoelectrolytes (COEs) represent a unique class of organic synthetic molecules, characterized by hydrophobic conjugated backbones and polar ionic pendants [14]. Both COEs and their polymer counterparts, conjugated polyelectrolytes (CPEs), have found applications as diagnostic tools in analyzing organelle structures, biological macromolecules, and pathogens [15,16,17,18,19]. Initial research on CPEs primarily revolved around the development of in vitro DNA sensors, leveraging the fluorescence resonance energy transfer (FRET) mechanism [15,16,17]. This was achieved through complexation with oppositely charged polyelectrolytes, such as DNA, facilitating the detection of specific single-stranded DNA [20] and distinguishing between single- and double-stranded DNA in the presence of intercalated dyes [17]. However, the use of CPEs has been constrained to in vitro settings due to their limited FRET sensitivity [16]. Additionally, the substantial polymer size of CPEs often resulted in their entrapment within lysosomes post-endocytosis, posing challenges in targeting subcellular compartments [21]. To address these limitations, monodisperse COEs with a precisely controlled structure were developed. These COEs, characterized by a consistent repeating unit in their conjugated backbones, exhibited electrostatic and hydrophobic interactions, enhancing their affinity and specificity for biological macromolecules [22,23,24,25,26,27]. The versatility of monodisperse COEs as a molecular platform was evident in their adaptability to various applications, achieved by altering the distribution and length of side chains and the architecture of conjugated backbone. In comparison to CPEs, COEs’ high quantum yield and well-defined chemical structure rendered them more effective for intracellular imaging and diagnostic applications [28].

As a iteration of monodisperse COEs, membrane-intercalating conjugated oligoelectrolytes (MICOEs) have been developed, featuring a linear hydrophobic backbone with hydrophilic polar pendants at both ends, mimicking the amphiphilic structure of lipid bilayers [14]. MICOEs have exhibited a diverse range of functionalities, including acting as antimicrobial agents [29], biocatalysts for electron transport across membranes [30], chemical stabilizers for membranes under environmental stress [23], photodynamic or photothermal therapeutic agents [31,32], and specific fluorescent markers for cellular or vesicle detection [25,26,27]. Currently, there is no research paper reporting the mechanism of COE binding with DNA and its applications in organelle localization. The binding of DNA and fluorescence localization of organelles have posed new challenges to the design of COE molecules. Discussions on the structural design of COE and its impact on molecular functions can be found in reference [14,33], where Zhou provides guiding insights into the development and functionality design of COE molecules, and Yan et al. extensively discuss two homologous series of COEs, including backbone length, structural modifications affecting membrane affinity, driving forces for membrane insertion, and the impact on toxicity. It is concluded that the backbone length of COE molecules has the greatest impact. For instance, a two-ringed COE2-2 has a weak driving force to spontaneously intercalate into cell membranes relative to other homologous COE molecules, and COE molecules with more than three benzene rings tend to be retained in the cell membrane. Therefore, the challenges for COE molecules in organelle localization and targeted DNA binding must balance the driving force for cell membrane penetration, avoid retention, and minimize size while fulfilling the functionality of a fluorescent probe. Considering these limitations and inspired by the intercalative properties of MICOEs, their well-defined structure, excellent aqueous solubility, high quantum yields, and large Stokes shifts, a hydrophobic backbone with three benzene rings was synthesized to effectively access the cellular interior and localize subcellular organelles and detecting intracellular DNA for the first time. In comparison to the previously reported COE2-3C molecule, the addition of six hydrophilic side chains reduces molecular toxicity and improves intracellular distribution [14,33].

In this study, we developed a new MICOE molecule, COE-S3, characterized by a backbone of three benzene rings and six hydrophilic ionic pendants at its terminals, designed for DNA quantification and cellular organelle localization. For the first time, COE-S3 demonstrated a specific affinity for double-stranded DNA (dsDNA). In live cells, COE-S3 localized to mitochondria, while in apoptotic cells, it targeted the nucleus for imaging. We also explored the intercalation binding of COE-S3 with dsDNA and examined how the length of the MICOE backbone influences its localization within intracellular organelles. The findings revealed that COE-S3, as a versatile fluorescent nucleic acid marker, holds significant potential for various applications in molecular biology.

## 2. Materials and Methods

### 2.1. Materials and Instruments

DNA, with a molecular weight of 15 million Daltons, was obtained from Sigma-Aldrich (Guangzhou, China, No. D4522). RNA from baker’s yeast and DNA were dissolved in pH = 7.4 Tris-buffer for titration assays. RNase (DNase-free) and DNase (RNase-free) were sourced from Takara. All other chemicals and solvents, used as received, were procured from Aladdin, Macklin, and Sigma. The Cells Counting Kit-8 assay (C0038) was purchased from Beyotime (Shanghai, China). Cell lines 4T1, H1299, Panc-1, and A549 were acquired from the Cell Culture Center at the Institute of Basic Medical Sciences, Chinese Academy of Medical Sciences, Beijing, China. Lyso-tracker probes were obtained from Abcam. The Mito-tracker and ER-tracker were purchased from Beyotime (Shanghai, China).

Nuclear Magnetic Resonance (NMR) spectra, including both ^1^H NMR and ^13^C NMR, were recorded using Bruker Avance spectrometers at 400 MHz and 500 MHz. Absorption and fluorescence emission spectra were measured using an Agilent Cary 60 UV-Vis spectrophotometer (Santa Clara, CA, USA) and a Hitachi F-4600 fluorescence spectrophotometer (Beijing, China), respectively. Cell viability assays were conducted using a BIO-TEK Synergy HT microplate reader (Santa Clara, CA, USA). The ZEISS LSM880 confocal laser scanning microscope was employed for cell imaging and organelle localization studies. Flow cytometric analyses were performed using a Beckman CytoFLEX flow cytometer (Shanghai, China).

### 2.2. Synthesis and Characterization of COE-S3

**Figure 1:**
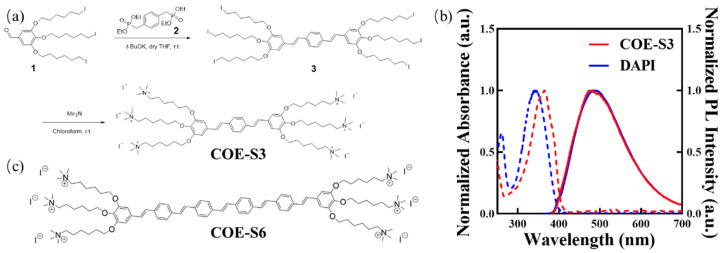
Chemical structures and photophysical properties. (**a**) Synthetic pathway of COE-S3. (**b**) Normalized absorption and photoluminescence (PL) spectra of COE-S3 following in vitro dsDNA treatment, with DAPI in aqueous solution serving as a control. (**c**) Chemical structure of the reference compound COE-S6. Adapted with permission from Ref. [23]. 2019, Cheng Zhou.

The COE-S3 molecule, featuring a backbone of three benzene rings and six terminal polar ionic pendants, was synthesized as detailed in Figure 1a, with comprehensive procedures and structural characterizations available in the Supporting Information (Figures S1–S3). Briefly, the oligo-phenylenevinylene backbone was constructed through the Horner–Wadsworth–Emmons reaction, where tetraethyl *p*-xylylenediphosphonate (Compound **2**) reacted with an excess of 3,4,5-tris((6-iodohexyl)oxy)benzaldehyde (Compound **1**, synthesized as per previous literature [23]) to form the neutral precursor **3**. Subsequently, COE-S3 was obtained as a yellow powder following quaternization with an excess of trimethylamine solution in a **3**/chloroform mixture, stirred at room temperature, and then purified by solvent removal under vacuum [23]. In aqueous solution, COE-S3 displayed maximum absorption at 366 nm and maximum emission at 476 nm, as shown in its UV–Vis absorption and fluorescence spectra (Figure 1b). Its spectral properties were similar to those of DAPI, and its molecular structure bore resemblance to the previously reported COE-S6 molecule (Figure 1c). Notably, COE-S3’s large Stokes shift (∆λ_max_ ≈ 100 nm) effectively minimizes self-absorption. The fluorescence quantum yield of COE-S3 in deionized water was determined to be approximately 0.80, using DAPI as the reference (QY_DAPI_ = 0.043) [34].

### 2.3. Spectroscopy Assays

The interactions between COE-S3 and nucleic acids were evaluated using spectroscopic assays, encompassing titration and circular dichroism experiments. In the titration experiments, DNA concentrations ranging from 0 to 10.67 mM were progressively added to a 1 μM COE-S3 solution. Following thorough mixing, the absorption and emission spectra were recorded, producing concentration-dependent curves centered at the absorption peak of 374 nm and the emission peak of 439 nm. For the circular dichroism studies, COE-S3 concentrations from 0 to 500 ng were introduced into a 0.33 mM DNA solution at room temperature. The absorption spectrum was subsequently recorded using a JASCO model J-815 spectropolarimeter. This analysis focused on identifying the characteristics of both positive and negative peaks, enabling a detailed assessment of the interaction dynamics between COE-S3 and the nucleic acids.

### 2.4. Molecular Docking Analysis

Molecular docking simulations between COE-S3 and nucleic acids were conducted using the AutoDock 4.2 tool. The compounds, designated as ligands, were initially drawn in ChemDraw and then converted to PDB format using Open Babel V3.1.0. The receptor structures for DNA (ID: 1P20, CGATCG) were sourced from the PDB database. Docking simulations were performed with default parameters set in the software. These parameters were further refined using the Lamarckian Genetic Algorithm (LGA) to determine the optimal ligand binding position, orientation, and conformation. A total of 300 random conformation positions were globally optimized, with the minimum binding energy serving as the criterion for evaluation.

### 2.5. Cell Imaging and Localizations

Cell culture was conducted under standard conditions at 37 °C in a humidified atmosphere with 5% CO_2_. The culture medium was supplemented with 10% (*v*/*v*) fetal bovine serum and 1% (*v*/*v*) penicillin-streptomycin. Cells grown on confocal petri dishes to a density of 70% were pre-treated as required for subsequent imaging. Fixed cells were prepared using 4% paraformaldehyde at room temperature for 10 min, followed by two PBS washes. Apoptotic cells were obtained from cells subjected to 72–96 h of serum starvation. For imaging, the treated samples were co-stained with COE-S3 and PI, each at 1 μM for 30 min, and then immediately imaged using confocal microscopy. In organelle localization experiments, live cells were pre-incubated with 1 μM COE-S3 (or COE-S6) for 4 h, followed by staining with Mito-tracker and Lyso-tracker probes for 45 min each. The colocalization coefficient was calculated post-confocal microscopy imaging, and the distribution of fluorescence curves was analyzed using Origin 8.0 software.

### 2.6. DNase and RNase Treatment

An affinity assay was performed using nuclease digestion treatments on stained, fixed cells. Three groups of pre-fixed H1299 cells underwent co-staining with COE-S3 and PI, each at a concentration of 1 μM for 30 min. This was followed by a double rinse with PBS. One group served as the control and was treated with 1 mL of PBS, while the other groups were incubated with 5 mg mL^−1^ nuclease for 4 h. After the incubation, the cells were washed twice with PBS and subsequently imaged using confocal microscopy, maintaining consistent laser parameters for all groups.

### 2.7. Cell Cycle Treatment

A total of 5 × 10^5^ cells, following a 24 h pre-incubation period with 100–200 nM PTX (with untreated cells as controls), were harvested by centrifugation at 1500 rpm for 3 min. These cells were then washed with chilled PBS, fixed in 70% ethanol for 18 h, and subjected to two additional cold PBS washes and centrifugation. Post-fixation, the cells were resuspended in 200 µL PBS containing DNase-Free RNase (50 µg mL^−1^) and incubated in a water bath at 37 °C for 30 min to digest RNA. After this, the cells were stained with PI, DAPI, and COE-S3 at final concentrations of 1 µM, respectively. Fluorescence measurements were performed using flow cytometry in both PB450 and ECD channels. Data from at least 3 × 10^4^ cells were collected using the CytoFLEX instrument and its accompanying software (Beckman Coulter, Shanghai, China). Analysis of the data was conducted using ModFit 5.0 software. For cell cycle analysis, the Dean–Jett–Fox model was applied to a minimum of 10,000 gated cells.

## 3. Results

### 3.1. Affinity to DNA and Interactions Mechanism

**Figure 2:**
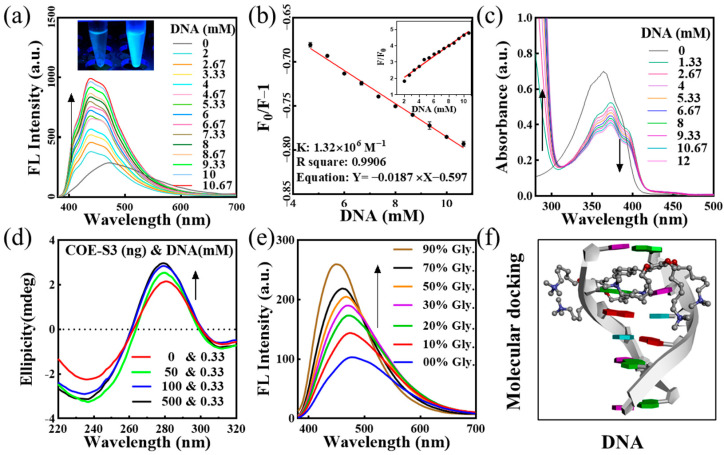
Analysis of COE-S3 and DNA interactions. (**a**) Fluorescence (FL) titration spectra of 1 μM COE-S3 in Tris-HCl buffer with varying concentrations of dsDNA (λ_ex_ = 355 nm). Inset: Emission fluorescence of COE-S3 with (right) and without (left) dsDNA treatments. (**b**) Fitted curve of the interaction based on the Scatchard equation, with (phosphate in DNA) ranging from 2 to 10.67 mM and 1 μM COE-S3. Inset: Graph depicting fluorescence variations at different DNA concentrations. (**c**) UV–Vis titration spectra of 1 μM COE-S3 in Tris-HCl buffer with incremental dsDNA concentrations. (**d**) Circular dichroism (CD) titration spectra of dsDNA with increasing COE-S3 concentrations. (**e**) Variations in the fluorescence emission spectra of 1 μM COE-S3 in glycerol/water solvent (λ_ex_ = 355 nm). (**f**) Molecular docking of COE-S3 with a DNA fragment using Discovery Studio tools (PDB ID: 1P20, CGATCG).

The interaction mechanism between probe molecules and nucleic acids is pivotal for targeted nucleus imaging. To elucidate the binding mode of COE-S3 molecules with nucleic acids, UV–Visible and fluorescence titration assays were conducted. COE-S3 demonstrated a distinct fluorescent response to dsDNA, evidenced by the bright blue fluorescence upon dsDNA addition (inset of Figure 2a). The fluorescence intensity of the emission spectra was directly proportional to DNA concentrations, showing a maximum blue shift (∆λ_max_ = 37 nm) with increasing DNA concentration (Figure 2a). The binding constant, calculated using the Scatchard equation, was found to be 1.32 × 10^6^ M^−1^, with an R^2^ value of approximately 0.9906, comparable to that of ethidium bromide (10^6^ M^−1^) [35,36]. Notably, the fluorescence intensity at 439 nm with 10.6 mM DNA was 4.9 times higher than that of COE-S3 alone (inset of Figure 2b), while no significant fluorescence enhancement was observed with RNA (Figure S4). The UV–Vis absorption spectra of the COE-S3-dsDNA complex (Figure 2c) showed a maximum absorption wavelength at 374 nm, with a slight red shift (∆λ_max_ ≈ 8 nm) and a corresponding decrease in absorbance at 374 nm upon increasing DNA concentration. The hyperchromicity at 280 nm, indicative of increased base stacking, suggested the disruption of double helix stability due to molecular interaction forces [37,38,39,40,41]. Further titration experiments results indicated that COE-S3 molecules had a greater affinity for DNA than RNA, and their binding mode with DNA was intercalative. The insertion of COE-S3’s planar π-delocalized backbone between base pairs restricted its free rotation, minimizing non-radiative transitions and resulting in a fluorescent response and hypochromic absorbance with DNA [36].

To confirm the intercalative binding of COE-S3 into dsDNA, circular dichroism (CD) spectroscopy was employed through assessing its impact on DNA’s secondary structure. The CD spectrum of free dsDNA solution exhibits two characteristic bands in the far-UV region (210–330 nm): a negative band at ~248 nm, indicative of right-handed helicity, and a positive band at ~278 nm, reflecting base stacking, consistent with the classical B-conformation of DNA [42,43]. Generally, the intercalative binding by small molecules altered the DNA secondary structure, resulting in changes to the intrinsic CD spectrum. In contrast, molecules interacting with DNA through electrostatic interactions or groove binding minimally perturb these bands [43,44,45]. The CD spectra, as shown in Figure 2d, revealed that increasing concentrations of COE-S3 significantly altered the positive peak (278 nm), with peak intensity correlating with COE-S3 concentration. This change suggested an alteration in the stacking interaction between base pairs (A-T and G-C). Concurrently, the decrease in the negative peak (248 nm) indicated an unfolding of the DNA helical structure. The CD spectrum of the COE-S3-DNA complex at various concentrations showed an enhanced Cotton effect, indicating conformational changes in DNA and intercalative binding in the presence of COE-S3 [46].

Viscosity tests, involving the gradual addition of glycerol to water/glycerol mixtures, were conducted to examine the relationship between the free rotational space of COE-S3 molecules and their fluorescence response. The fluorescence intensity of COE-S3 was found to be directly proportional to the solution’s viscosity (Figure 2e), supporting the hypothesis of an intercalative binding mode. Intercalation binding to DNA typically increased the mixture’s viscosity due to the enlargement of gaps between adjacent base pairs. In contrast, electrostatic and groove binding modes had a minimal impact on viscosity [47,48].

To further support our hypothesis, we conducted theoretical molecular docking simulations for DNA/RNA and COE-S3 by using Discovery Studio software. The results, shown in Figure 2f, utilized a DNA model from the PDB database (PDB ID: 1P20, CGATCG), with the CDOCKER energy for intercalation calculated at –6.91 kcal mol^−1^. The RNA complex simulation results as depicted in Figure S5 indicated groove binding, with the luminescent group of the molecule being enveloped within the groove, providing a plausible explanation for the observed lack of fluorescence enhancement with RNA. In summary, our findings suggest that the material can indeed bind to both DNA and RNA, but only exhibits fluorescence enhancement when interacting with dsDNA, hence showing affinity towards DNA.

### 3.2. Co-Localization in Cell Organelles

**Figure 3:**
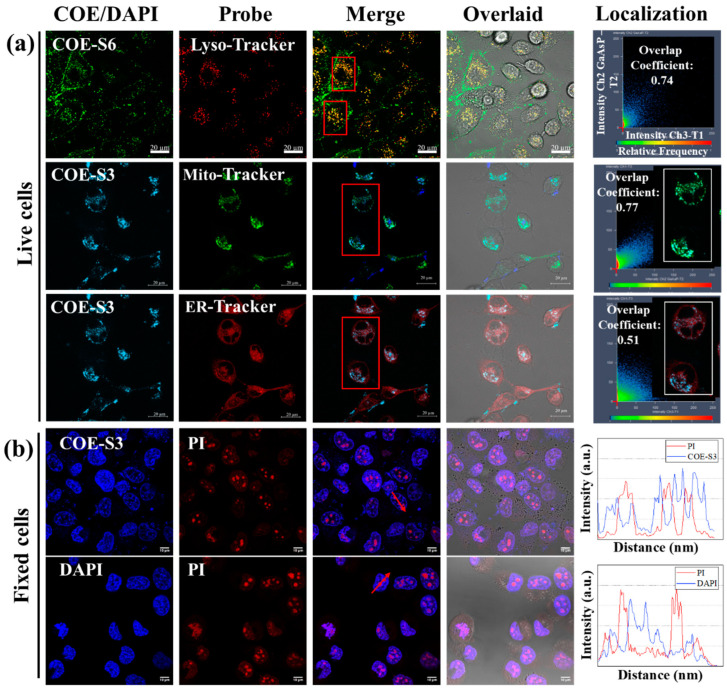
Intracellular localization and nucleus imaging with COE-S3. (**a**) Confocal microscopy images of live Panc-1 cells illustrating the colocalization of COE compounds with commercial organelle probes. Cells were pre-incubated with 1 μM COE-S6 or COE-S3 for 4 h, followed by staining with 10 μM organelle-specific probes in DMEM for 45 min. The fluorescence colocalization coefficients between COE and probe were calculated using ZEN software for regions of interest (inside the red box). Scale bars are 20 µm. (**b**) Confocal microscopy images of fixed H1299 cells co-stained with 1 μM COE-S3 (or DAPI) and 1 μM PI for 30 min, including intensity profiles across regions of interest (with arrows). Scale bars are 10 µm. Excitation and emission wavelengths: COE-S3 and DAPI (λ_ex_ = 405 nm, λ_em_ = 430–490 nm); COE-S6 (λ_ex_ = 488 nm, λ_em_ = 500–540 nm); Mito-tracker green (λ_ex_ = 488 nm, λ_em_ = 516 nm); ER-tracker red and Lyso-tracker red (λ_ex_ = 532 nm, λ_em_ = 560–600 nm).

The spectral analysis of COE-S3’s interaction with nucleic acids revealed a specific fluorescence response to DNA, enabling sensitive in vitro DNA detection. This finding spurred interest in the targeted localization of COE-S3 within cellular organelles. The localization and distribution of the COE-S3 molecules in cells were investigated through co-localization imaging with organelle-specific fluorescent probes. Additionally, the length of membrane-intercalated COE has been reported to regulate its effect on lipid bilayers, which can range from membrane disruption and permeation to stabilization. No relevant studies have reported the regulation of COE molecular length in organelle localization after crossing the cell membrane barrier. Thus, an elongated molecule, COE-S6, with identical hydrophilic chains, only differing in the six repeated units on the core backbone was utilized to compare with COE-S3 to investigate the regulation of molecular length. As shown in Figure 3a, after a 4 h pre-incubation period with COE materials, the intracellular green fluorescence of COE-S6 overlapped with the red fluorescence of the Lyso-tracker probe, while the intracellular blue fluorescence of COE-S3 overlapped with the green fluorescence of the Mito-tracker probe and the red fluorescence of the ER-tracker probe, yielding overlap coefficients of 0.74, 0.77 and 0.51, respectively. To clearly observe the organelle localization, COE-S3 staining was treated with light blue pseudo-colors in live cells. These results suggest that COE-S6 localizes to the lipid membrane and lysosomes, whereas COE-S3 targets mitochondria in live cells.

In order to illustrate the difference in the affinity of molecular lengths for intracellular substances, fixed cell staining experiments were carried out. In fixed cells, COE-S3 molecules were predominantly found in the nucleus, emitting blue fluorescence, while COE-S6 molecules, emitting green fluorescence, localized to the cell membrane under the same treatment conditions (Figure S6). This result indicates that the elongated COE-S6 molecule primarily integrates into the membrane in fixed cells and the COE-S3 molecule is mainly confined to the nucleus, and exhibits strong affinity for intracellular DNA. Collectively, COE-S6 molecules exhibit strong selectivity for the cell membrane, and are confined to lysosomes in live cells. In contrast, the shortened COE-S3 molecules display a strong affinity for intracellular DNA, distributing within the mitochondria in live cells and concentrating in the cell nucleus in dead cells. The disparity in organelle localization is attributed to the influence of molecular length. Under identical side-chain conditions, longer molecules are confined to lysosomes, unable to escape their limitations, while shorter molecules, due to electrostatic attraction, bind to mitochondrial DNA, enabling mitochondrial imaging. The different selectivity of COE-S6 and COE-S3 can be attributed to their respective matching with membrane size and DNA structure size.

To validate the specific affinity of COE-S3 for intracellular DNA in the nucleus, the lung cancer cell line H1299 was employed as a model. Cells were fixed using a 4% formaldehyde solution and co-stained with 1 μM COE-S3 and Propidium Iodide (PI), a commercial nuclear dye, in darkness at room temperature for 30 min. For comparison, DAPI and PI were applied under identical conditions. PI is a non-permeant red-fluorescent dye that binds to both DNA and RNA, whereas DAPI selectively binds to DNA. As illustrated in Figure 3b, the merged images displayed distinct nuclear morphology with strong blue fluorescence from COE-S3 pervading the nuclei, except in areas showing intense red signals from PI, identified as nucleoli, which were rich in ribosomal RNA [37,49]. This pattern was similarly observed in DAPI-stained cells, and the staggered fluorescence peak curves further confirmed the higher affinity of COE-S3 and DAPI for intracellular DNA over RNA. To ensure DNA-selective localization, the fluorescence co-localization coefficient of PI and COE-S3 was calculated to be 0.93 following RNase treatment, minimizing fluorescence interference from RNA (Figure S7). The nuclear staining patterns of COE-S3 were also consistent across other cell lines (Figure S8 in Supporting Information). The amphipathic nature of the COE-S3 molecule, coupled with its shortened backbone structure, conferred specific DNA affinity, enabling it to evade lysosomal entrapment. In live cells, COE-S3 predominantly localized to mitochondria due to weak electrostatic interactions with the mitochondrial membrane potential and its affinity for mitochondrial DNA, facilitating mitochondria imaging. Conversely, in fixed cells, COE-S3 targeted DNA-rich nuclei for nuclear imaging, as organelles lose functionality in the presence of paraformaldehyde.

### 3.3. Nuclease Digestion Assay and Nucleus Imaging

**Figure 4:**
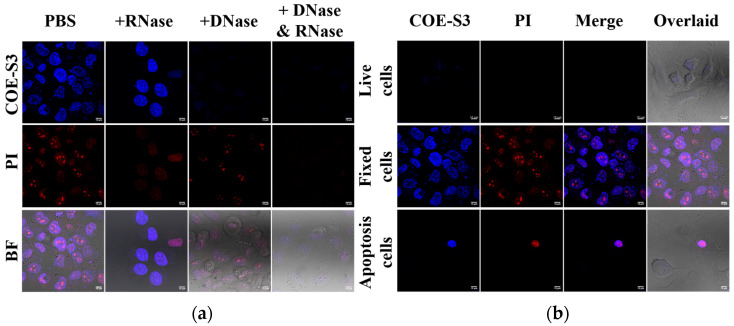
Nuclease digestion and nucleus imaging assays. (**a**) Nuclease digestion assay in fixed H1299 cells stained with 1 μM COE-S3 (or DAPI) and 1 μM PI. (**b**) Confocal nucleus imaging in live, fixed, and apoptotic cells, co-stained with 1 μM COE-S3 and 1 μM PI. Excitation and emission wavelengths: COE-S3 and DAPI (λ_ex_ = 405 nm, λ_em_ = 430–490 nm); PI (λ_ex_ = 532 nm, λ_em_ = 560–600 nm). Scale bars are 10 µm.

To confirm the specific binding of COE-S3 to intracellular DNA and to rule out nonspecific interactions with RNA, a nuclease digestion assay using both RNase and DNase was performed. This assay aimed to validate the selective affinity of COE-S3 for DNA within cells. Cells pre-stained with COE-S3 were treated separately with PBS, DNase, and RNase. Remarkably, the fluorescence intensity of COE-S3 markedly diminished following DNase treatment, indicating DNA degradation, whereas RNase treatment, which degrades RNA, did not affect the fluorescence intensity (Figure 4a). These findings were corroborated in control experiments: DNase treatment reduced the blue fluorescence of DAPI, a DNA-specific dye, and RNase treatment eliminated the deep red fluorescence of PI from the nucleolus, as shown in Figure S9. The pronounced selectivity of COE-S3 for intracellular DNA eliminates the need for RNase pre-treatment, a step often required for commercial DNA probes such as ethidium bromide, thiazole orange homodimer, and SYTO dyes [38].

COE-S3 exhibited a pronounced DNA-specific affinity, enabling efficient nuclear imaging in fixed cells within a brief duration. To assess the functionality of COE-S3 in nuclear imaging under various conditions, different cell treatments were applied to obtain live cells, spontaneously apoptotic cells, and fixed cells, as previously described (Figure 4b). The results demonstrated COE-S3’s capability to differentiate cell viability via nuclear visualization. In live cells, the membrane-permeable COE-S3 showed faint fluorescence and failed to outline the nucleus within 30 min. In contrast, fixed cells displayed a well-defined nucleus with intense fluorescence from both COE-S3 and PI. This distinction was more pronounced in apoptotic cells, where cell viability was discernible through fluorescent labeling: live cells exhibited no fluorescence, whereas the nuclei of apoptotic cells, appearing shrunken, were brightly labeled. A plausible explanation is that in living cells, only a limited quantity of COE-S3 molecules could penetrate the cell membrane within 30 min, predominantly localizing in the mitochondria. However, when cell viability was compromised, the loss of membrane integrity and mitochondrial function facilitated increased binding of COE-S3 molecules to DNA within the nucleus, resulting in augmented fluorescence. These findings underscore the potential of COE molecules as a valuable tool for assessing cell viability through nuclear fluorescence, particularly in cells with compromised membrane integrity.

### 3.4. Photostability and Cytotoxicity Evaluation

Photostability is a critical parameter for bioimaging applications. To evaluate this, the time-dependent fluorescence intensity of intracellular COE-S3 was analyzed through repeated confocal microscopy scans. Remarkably, intracellular COE-S3 exhibited robust photostability, retaining 85% of its initial fluorescence intensity even after 200 laser confocal scans, as demonstrated in Figure S10. This sustained fluorescence signal underscores the potential of COE-S3 for long-term imaging applications.

The biosafety of COE-S3 molecules is paramount for their biomedical application. To assess cytotoxicity, we assessed the cell viability of COE-S3 materials in four cancer cell lines (PANC-1, 4T1, Hela and H1299) and two normal cell lines (human umbilical vein endothelial cells (HUVEC), normal embryonic fibroblast cell line (NIH/3T3)) for 24 h. The results (refer to Figure S11) indicated that the cell viability remained above 91.45 ± 2.2% even at a high concentration of 20 µM of COE-S3 among all cell lines. These data suggest that COE-S3 exhibits low toxicity, affirming their suitability as safe intercalative probes for nuclear imaging. In our study, the damage to cells at a concentration of 1 micromole for 30 min was deemed negligible, considering the applications of imaging and quantitative detection.

### 3.5. Quantitative Detection of Intracellular DNA

**Figure 5:**
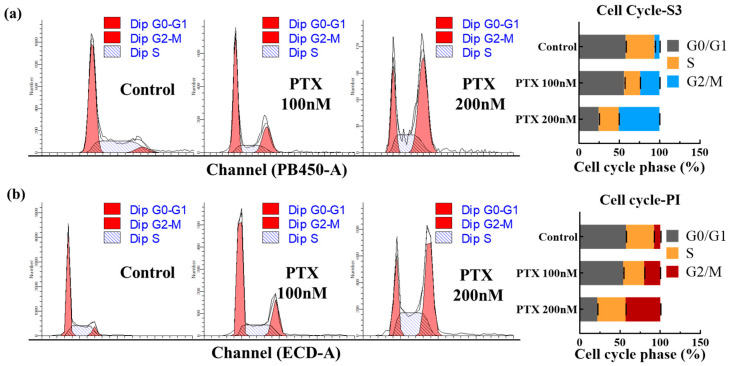
Cell cycle analysis with COE-S3 and PI. (**a**) Quantitative assessment of cell cycle phases in three groups using COE-S3. (**b**) Quantitative assessment of cell cycle phases in three groups using PI. Analysis includes normal cells, and cells treated with 100 nM and 200 nM PTX over 24 h. Data processed using ModFit 5.0 software. Results are presented with mean standard deviation (±S.D.).

The cell cycle assay is a vital method for assessing cell growth status, focusing on variations in the total amount of intracellular DNA [49]. The cell cycle, a sequence of events from one division to the next, is categorized into the interphase and division phase [50]. The interphase includes three stages: the pre-DNA synthesis phase (G1), DNA synthesis (S phase), and post-DNA synthesis (G2 phase) [51,52]. In this study, COE-S3, known for its specific affinity to dsDNA, was employed for cell cycle analysis via flow cytometry, demonstrating its utility in quantitative DNA analysis. We utilized two cell populations for this analysis: normal A549 cells and A549 cells treated with varying concentrations of paclitaxel (PTX), a chemotherapeutic agent that disrupts cell microtubules, inhibiting mitosis and leading to cell death [53]. PTX treatment resulted in an increased proportion of cells in the G2/M phase and a rise in 4N content, indicative of mitosis inhibition. As shown in Figure 5, COE-S3 effectively delineated the cell cycle stages in both PTX-treated and untreated cells. For comparison, commercial fluorescent dyes DAPI and PI were used as controls, with the histogram analysis results presented in Figure S12. Notably, a 24 h treatment with 200 nM PTX led to a higher proportion of cells in the G2/M phase and a reduction in the G0/G1 phase. The G2/M phase proportion escalated with increasing PTX concentration. Significantly, the findings with 1 µM COE-S3 treatment aligned with those obtained using commercial dyes, accurately quantifying intracellular DNA and providing insights into cell growth status and nuclear bioimaging.

## 4. Conclusions

In conclusion, COE-S3, characterized by its planar structure with three benzene rings in the oligophenylenevinylene backbone, was designed to explore its interaction with double-stranded DNA for targeted intracellular localization. This study confirmed COE-S3’s ability to intercalate into dsDNA, resulting in enhanced base stacking and fluorescence, as evidenced by UV–Vis, fluorescence, and CD spectroscopy. The fluorescence intensity of COE-S3 linearly increased with DNA concentration, showing at least a 4.9-fold enhancement. Nuclease digestion assays (RNase and DNase) established COE-S3’s specific DNA selectivity within cells. Co-localization imaging demonstrated that amphiphilic COE-S3 preferentially associates with DNA in the mitochondria of living cells and nuclei of apoptotic cells. Rapid staining with COE-S3 enabled differentiation between living and apoptotic cells in mixed cultures. Additionally, cell cycle analysis using flow cytometry on PTX-treated cells revealed that 1 µM COE-S3 treatment accurately quantified intracellular DNA, providing insights into cell growth status. Overall, COE-S3 emerges as a multifaceted and valuable tool for measuring intracellular DNA, nuclear bioimaging, and indicators of dead cells, offering significant potential for cancer research and drug development. In the future, the COE structure can be improved by changing the optical properties by introducing of donor–acceptor fragments or cross-conjugated backbones, modifying the side chains with functional groups, and other means combined with theoretical simulations to meet high-precision and targeted sensing applications.

## Data Availability

Data are contained within the article.

## Supplementary Materials

The following supporting information can be downloaded at: https://www.mdpi.com/article/10.3390/bios14020105/s1, Figure S1: ^1^H NMR spectrum of compound 3 in deuterated chloroform; Figure S2: ^13^C NMR spectrum of compound 3 in deuterated chloroform; Figure S3: ^1^H NMR spectrum of compound COE-S3 in deuterated DMSO; Figure S4: Fluorescence responses of COE-S3 (1 µM) to RNA at different concentrations in Tris–HCl buffer. [RNA] = 0–9 nM; Figure S5: Molecular docking of COE-S3 with an RNA fragment using Discovery Studio tools (PDB ID: 2LWK, GAGUAGAAACAAGGCU); Figure S6: Intracellular localization of COE-S6 molecules and COE-S3 molecules (1 µM) in fixed cells; Figure S7: The fluorescence co-localization mapping and image of PI and COE-S3 after RNase treatment.; Figure S8: COE-S3 staining experiments in different cell lines; Figure S9: Fluorescent intensity of different conditions of S3, DAPI and PI in nuclease digestion assay; Figure S10: The photostability evaluation of COE-S3; Figure S11: Cytotoxicity of COE-S3 at various concentrations in four cell lines including: H1299, HeLa, 4T1, and Panc-1 cells over 24 h; Figure S12: Cell cycle histogram analysis obtained using flow cytometry. Normal A549 cells (i–iii) and 100nM PTX-treated cells (iv–vi) over 24 h.
