# Supplementary material for: Conjugated Oligoelectrolyte with DNA Affinity for Enhanced Nuclear Imaging and Precise DNA Quantification
Source: Biosensors (Basel). 2024 Feb 18;14(2):105. doi: 10.3390/bios14020105 (PMC10887168; doi:10.3390/bios14020105)
Supplement: Supplementary file 1 [file biosensors-14-00105-s001.zip › biosensors-2839973-supplementary.pdf]

Supporting Information

Conjugated Oligoelectrolyte with DNA Affinity for Enhanced Nuclear Imaging and Precise DNA Quantification

Xinmeng Zhang [1,2], Cheng Zhou [3,*], Jianxun Hou [1], Gang Feng [4], Zhourui Xu [4], Yonghong Shao [2], Chengbin Yang [4] and Gaixia Xu [4,*]

Supporting Information List:

Figure S1. ^{1}H NMR spectrum of compound 3 in deuterated chloroform.

Figure S2. ^{13}C NMR spectrum of compound 3 in deuterated chloroform.

Figure S3. ^{1}H NMR spectrum of compound COE-S3 in deuterated DMSO.

Figure S4. Fluorescence responses of COE-S3 (1μM) to RNA at different concentrations in Tris–HCl buffer. [RNA] = 0-9 nM.

Figure S5. Molecular docking of COE-S3 with an RNA fragment using Discovery Studio tools (PDB ID: 2LWK, GAGUAGAAACAAGGCU), and the CDOCKER energy of COE-S3 with RNA.

Figure S6. Intracellular localization of COE-S6 molecules and COE-S3 molecules (1μM) in fixed cells.

Figure S7. The fluorescence co-localization mapping and image of PI and COE-S3 after RNase treatment.

Figure S8. COE-S3 staining experiments in different cell lines.

Figure S9. Fluorescent intensity of different conditions of S3, DAPI and PI in nuclease digestion assay.

Figure S10. The photostability evaluation of COE-S3.

Figure S11. Cytotoxicity of COE-S3 at various concentrations in four cell lines including: H1299, HeLa, 4T1, and Panc-1 cells over 24 h.

Figure S12. Cell cycle histogram analysis obtained from flow cytometry. Normal A549 cells (i-iii) and 100nM PTX-treated cells (iv-vi) over 24 h.

Synthesis:

The 3,4,5-tris((6-iodohexyl)oxy)benzaldehyde (compound **1**) was synthesized according to previous literature. The chemicals, including trimethylamine solution in methanol (3.2 M), tetraethyl *p*-xylylenediphosphonate (compound **2**), potassium *tert*-butoxide, dry tetrahydrofuran (THF), chloroform, were purchased from Sigma-Aldrich, Fisher or TCI chemicals and used directly without further purification. The ^{1}H and ^{13}C NMR spectra were measured on Bruker AV 400 or AV 500 spectrometers in deuterated chloroform or dimethyl sulfoxide (DMSO) at room temperature. Chemical shifts were reported as δ values (ppm) relative to an internal tetramethylsilane (TMS) standard.

Synthesis of 1,4-bis((*E*)-3,4,5-tris((6-iodohexyl)oxy)styryl)benzene (Compound 3):

3,4,5-Tris((6-iodohexyl)oxy)benzaldehyde (compound **1**) (0.72 g, 0.93 mmol), tetraethyl *p*-xylylenediphosphonate (compound **2**) (0.14 g, 0.37 mmol), and 10 mL dry THF were added to a round flask under the protection of nitrogen atmosphere. Then, potassium *tert*-butoxide (0.04 g, 0.37 mmol) was added into the reaction mixture under stirring. After a 16 h reaction at room temperature, the reaction mixture was poured into water, and extracted with chloroform. The transparent organic phase was dried over Na_2SO_4 and then we removed the organic solvent using a rotary evaporator. The crude product was purified with column chromatography using hexane and dichloromethane as eluents, and then the product was obtained as a yellow solid (0.53 g, 87 % yield). ^{1}H NMR (400 MHz, Chloroform-*d*) δ 7.58 (br, 4H), 7.16 – 7.03 (m, 4H), 6.82 (s, 4H), 4.19 – 4.11 (m, 8H), 4.10 – 4.04 (m, 4H), 3.38 – 3.28 (m, 12H), 2.04 – 1.82 (m, 24H), 1.66 – 1.54 (m, 24H). ^{13}C NMR (101 MHz, $CDCl_3$) δ 153.53, 138.49, 136.91, 133.07, 128.95, 127.81, 127.07, 105.57, 73.60, 69.26, 33.94, 33.79, 30.79, 30.60, 30.47, 29.60, 25.50, 25.46, 7.52, 7.33.

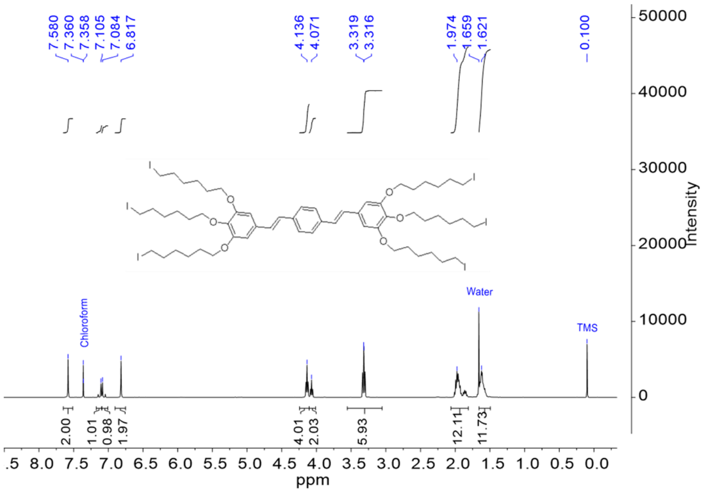

Figure S1. ^{1}H NMR spectrum of compound **3** in deuterated chloroform.

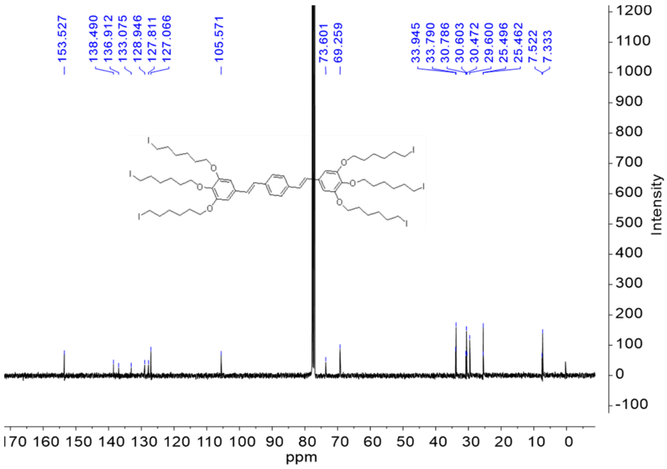

Figure S2. ^{13}C NMR spectrum of compound **3** in deuterated chloroform.

Synthesis of targeting compound (COE-S3):

A single-neck round flask was charged with compound **3** (253 mg, 0.154 mmol) and chloroform (10 mL) under nitrogen atmosphere. After the compound **3** dissolved, 0.5 mL trimethylamine solution in methanol (3.2 M) was added

into the reaction mixture and stirred at room temperature for 16 h. Then, a large excess of trimethylamine solution in methanol (5 mL, 3.2 M) was added into the reaction mixture and stirred for another 24 h. The solvent was removed via rotary evaporation and dried in a vacuum. The final product was obtained as a yellow solid (276 mg, 90 % yield). ^{1}H NMR (500 MHz, Chloroform-*d*) δ 7.59 (br, 4H), 7.22 (br, 4H), 6.93 (br, 4H), 4.10 – 3.98 (m, 8H), 3.94 – 3.85 (m, 4H), 3.42 – 3.26 (m, 12H), 3.07 (s, 27H), 1.83 – 1.64 (m, 24H), 1.58 – 1.45 (m, 12H), 1.41 – 1.28 (m, 12H).

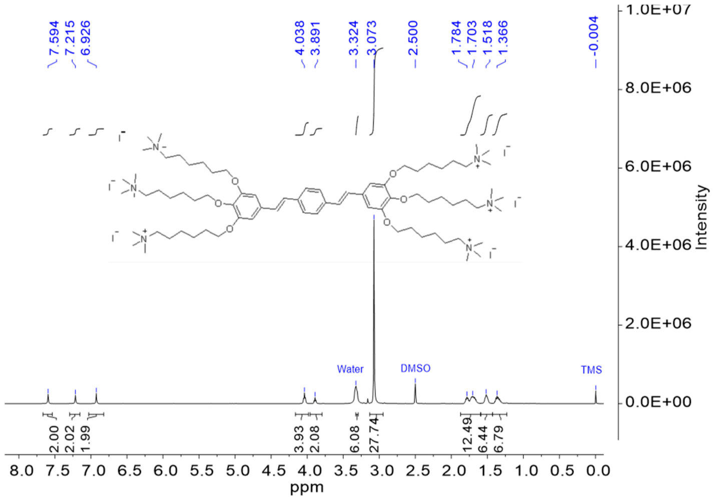

Figure S3. ^{1}H NMR spectrum of compound **COE-S3** in deuterated DMSO.

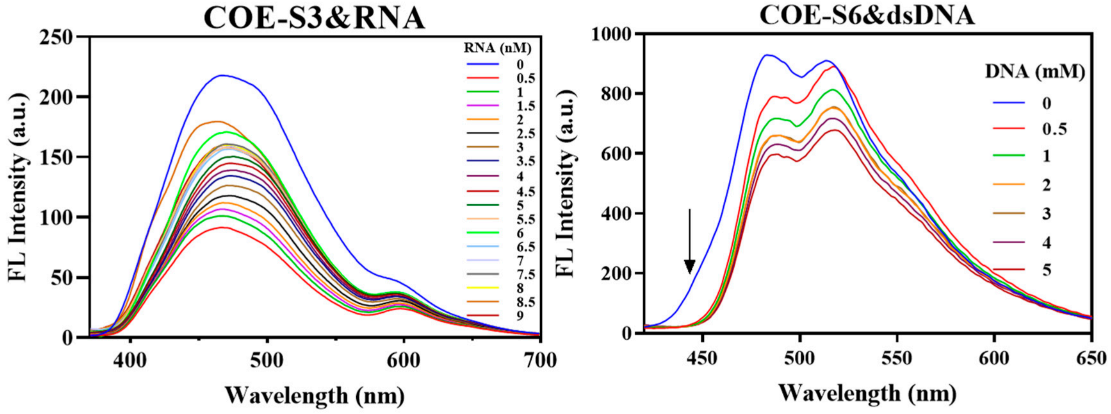

Figure S4. Fluorescence responses of COE-S3 (1μM) to RNA at different concentrations in Tris–HCl buffer. [RNA] = 0-9 nM.

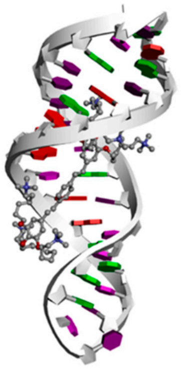

Cdocker Energy

RNA		average
1	-9.54	-8.71
2	-9.41	
3	-8.93	
4	-8.63	
5	-8.54	
6	-8.41	
7	-8.15	
8	-8.07	

Figure S5. Molecular docking of COE-S3 with an RNA fragment using Discovery Studio tools (PDB ID: 2LWK, GAGUA-GAAACAAGGCU), and the CDOCKER energy of COE-S3 with RNA.

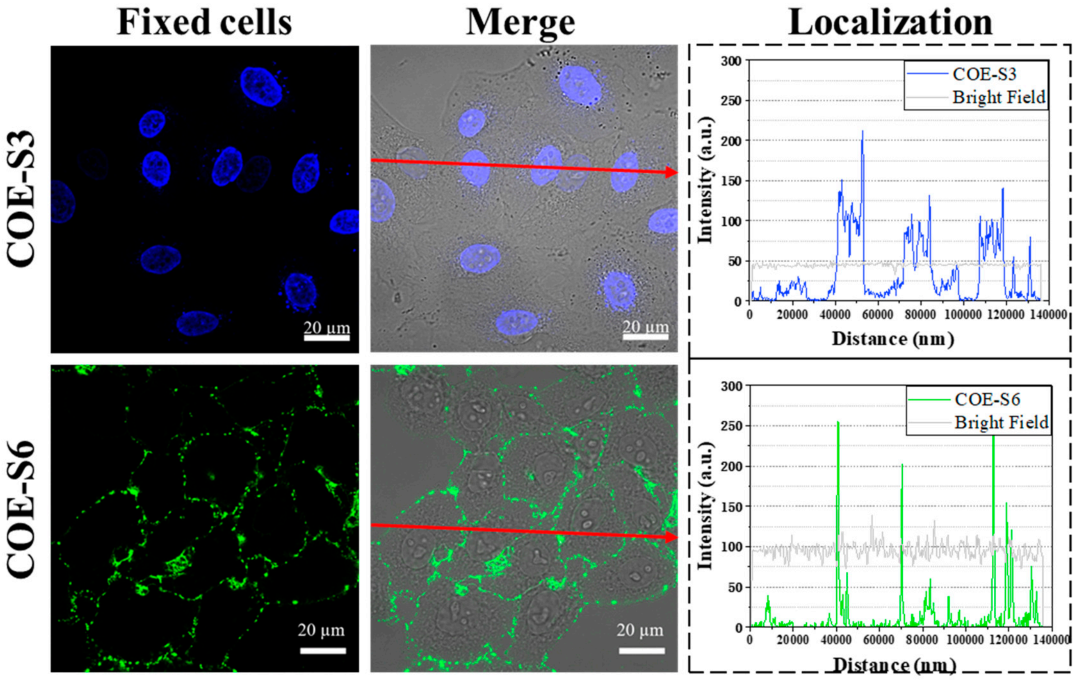

Figure S6. Intracellular localization of COE-S6 molecules and COE-S3 molecules (1µM) in fixed cells.

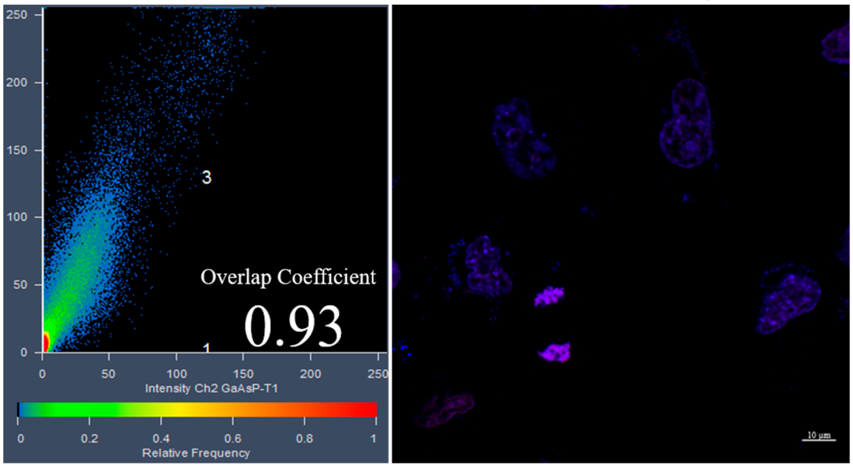

Figure S7. The fluorescence co-localization mapping and image of PI and COE-S3 after RNase treatment.

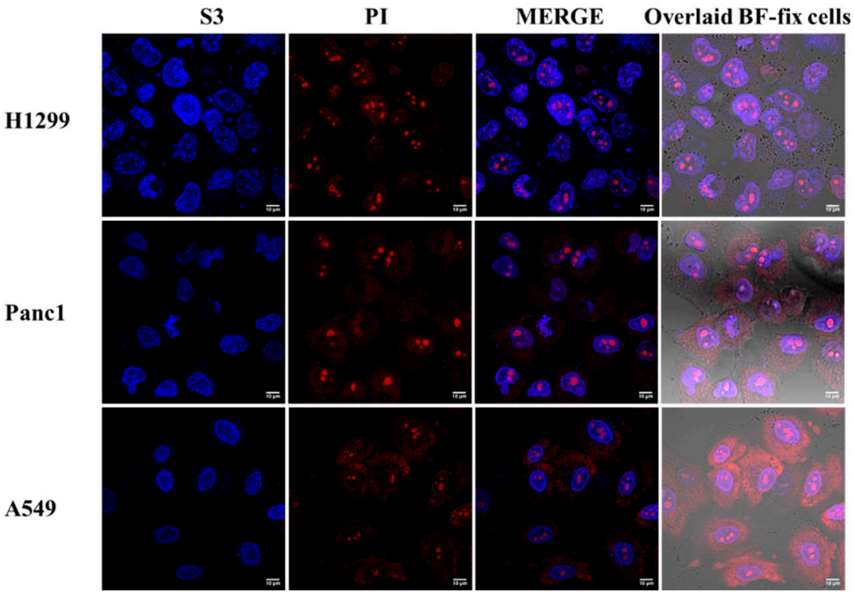

Figure S8. COE-S3 staining experiments in different cell lines.

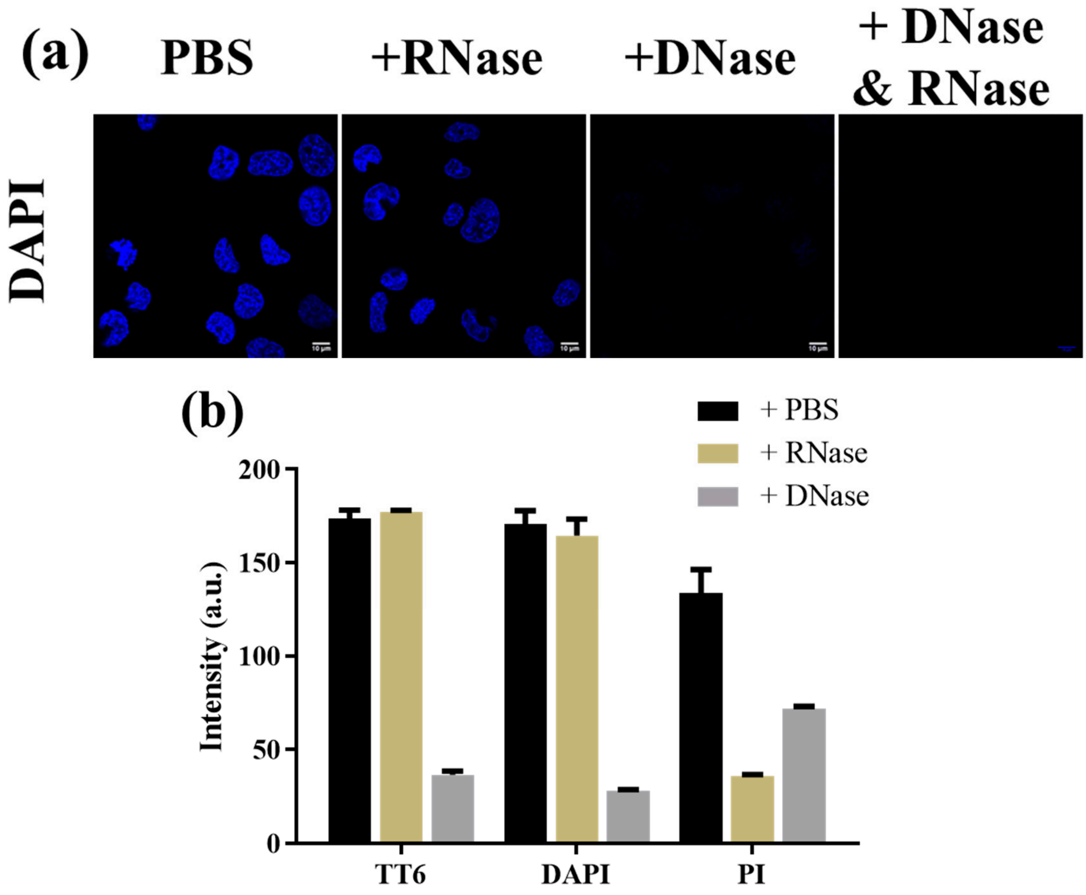

Figure S9. Fluorescent intensity of different conditions of S3, DAPI and PI in nuclease digestion assay.

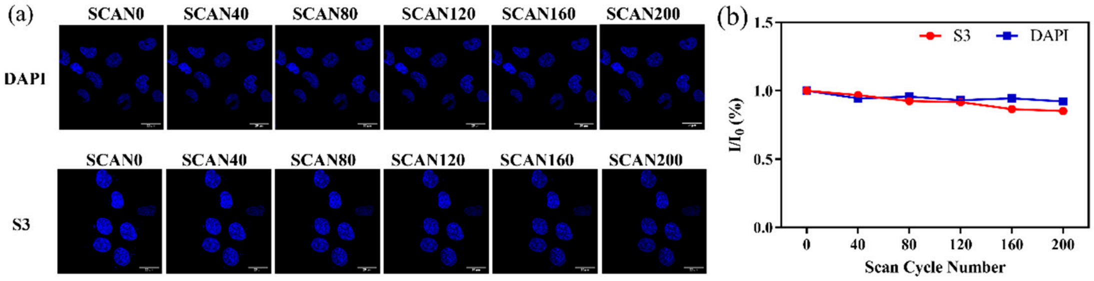

Figure S10. The photostability evaluation of COE-S3. (a) Fluorescence images of COE-S3 and DAPI (1μM) in pre-fixed H1299 cells. Scale bar :20 μm. (b) Fluorescence intensities of COE-S3 and DAPI in pre-fixed H1299 cells under 200 times scanning by confocal microscopy.

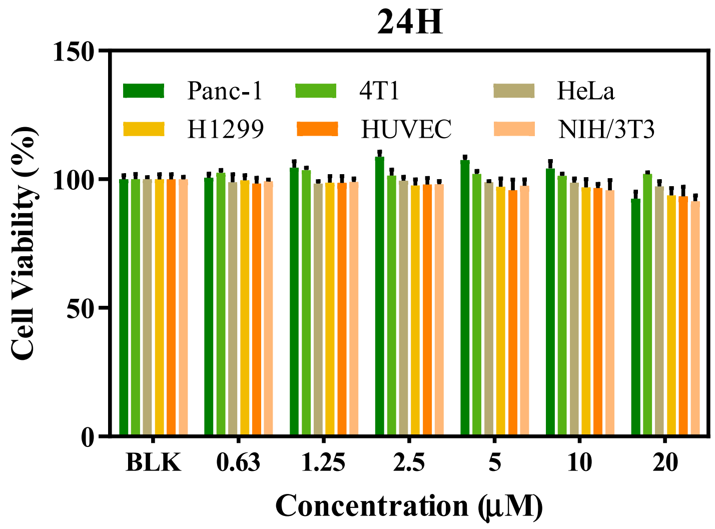

Figure S11. The cytotoxicity of COE-S3 was evaluated using the CCK-8 assay on six cell lines. The cells were incubated for 24h with different concentrations of COE-S3. Blank cells were untreated. The results are represented as means ± SD, n = 4.

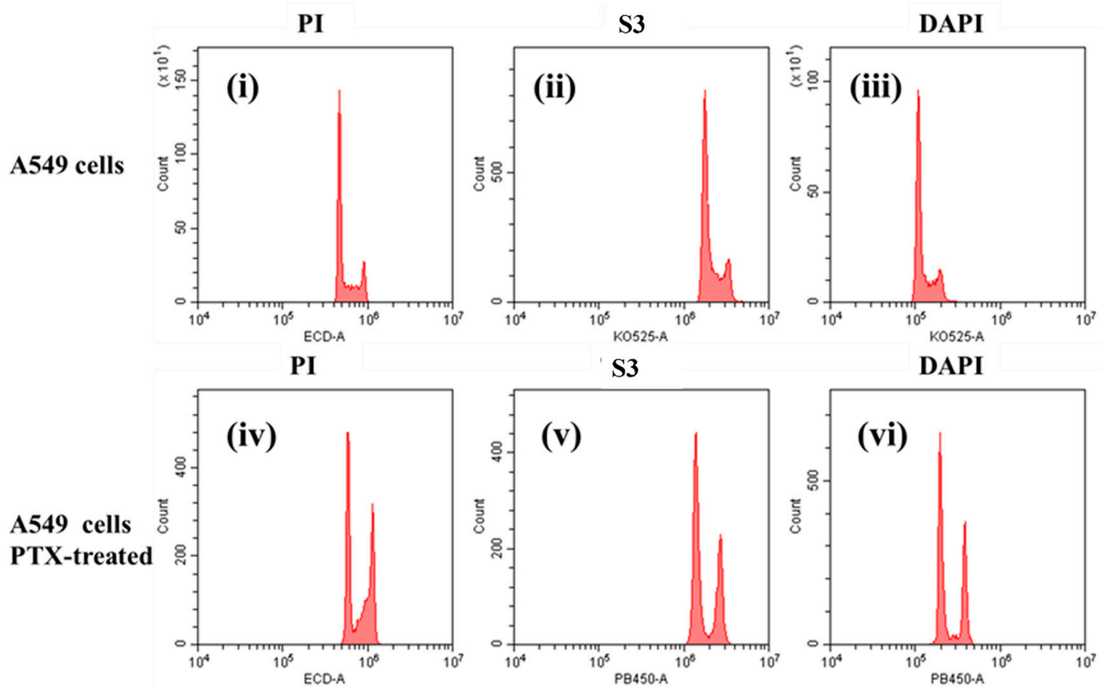

Figure S12. Cell cycle histogram analysis obtained using flow cytometry. Normal A549 cells (i-iii) and 100nM PTX-treated cells (iv–vi) over 24 h.
